# Pulmonary Barotrauma in COVID-19 Patients: Experience From a Secondary Care Hospital in Oman

**DOI:** 10.7759/cureus.26414

**Published:** 2022-06-29

**Authors:** Rasathurai Kajenthiran, Manish Kumar Tiwary, Ashok Lal, Jacob Paul, Faisal Al Sawafi, Yogesh Manhas, Ajay Yadav, Zaina Al Harthi, Abhijit Nair

**Affiliations:** 1 Anesthesiology, Ibra Hospital, Ibra, OMN; 2 Critical Care, Ibra Hospital, Ibra, OMN; 3 Respiratory Medicine, Ibra Hospital, Ibra, OMN

**Keywords:** covid-19, pulmonary barotrauma, morbidity and mortality, invasive mechanical ventilation, ards (acute respiratory distress syndrome)

## Abstract

Background

During the COVID-19 pandemic caused by severe acute respiratory syndrome coronavirus 2 (SARS-CoV-2), many patients developed pulmonary barotrauma either self-inflicted or ventilator-induced. In pulmonary barotrauma, air leaks into extra-alveolar tissue resulting in pneumomediastinum, subcutaneous emphysema, pneumothorax, and pneumoperitoneum.

Methods

After obtaining institutional approval, we retrospectively reviewed data from March 1, 2021, to September 31, 2021. Being a retrospective study, informed consent was not applicable. Patient data were collected from the Al Shifa patient information portal, which is an electronic medical record system available to all hospitals in the Ministry of Health, Oman. After identifying patients with pulmonary barotrauma, the following details were recorded and entered into an Excel sheet (Microsoft Corporation, Albuquerque, New Mexico) and a database was created, which contained the following: age, sex, smoking history, comorbidities, type, location, mode of barotrauma, mode of ventilation, length of intensive care unit (ICU) stay, interventions performed, and overall outcome (survived/deceased).

Results

A total of 529 patients with COVID-19 pneumonia were admitted from March 2021 to September 2021 to the ICU. Twenty-eight patients developed barotrauma of variable severity and required interventions like the placement of intercostal drains. Out of 28, five patients developed spontaneous barotrauma, 14 patients had barotrauma after initiation of non-invasive ventilation, and nine patients had barotrauma as a result of invasive ventilation. The median number of days in the ICU was 19.5 (interquartile range: 12.5-26.5). Of the 28 patients, eight patients survived and were discharged from the hospital.

Conclusion

In this single-center, retrospective study at a secondary care hospital in Oman, we described our experience with patients who suffered pulmonary barotrauma during their ICU admission. We have also presented the incidence of spontaneous versus ventilator-induced barotrauma, the length of stay of these patients, the outcomes in terms of survival or death, the need for tracheostomy, secondary infections, and interventions performed as indicated.

## Introduction

Pulmonary barotrauma refers to alveolar rupture due to increased transalveolar pressure (the difference between the alveolar pressure and the pressure in the adjacent interstitial space). This leads to air leakage into extra-alveolar tissue, which leads to pneumomediastinum, subcutaneous emphysema, pneumothorax, and pneumoperitoneum [1,2]. During the COVID-19 pandemic caused by severe acute respiratory syndrome coronavirus 2 (SARS-CoV-2), clinicians encountered many patients who developed barotrauma of varying types and severity during invasive/non-invasive ventilation, and even during spontaneous breathing in some cases [3-6].

The high respiratory drive present in patients with underlying severe lung injury leads to the intense respiratory effort that exacerbates the underlying lung injury and manifests as pulmonary barotrauma. This is referred to as “patient-self-inflicted lung injury” (P-SILI) [7]. Pulmonary barotrauma leads to prolonged intensive care unit (ICU) stay, increases the duration of mechanical ventilation, leads to difficult weaning from mechanical ventilation, and increases morbidity and mortality [8,9]. The results of the observational study by Kahn et al. mentioned a mortality rate of 56% in the presence of barotrauma in patients with COVID-19 acute respiratory distress syndrome (ARDS) when compared to 36% without barotrauma [10]. When barotrauma is suspected and confirmed by imaging (chest radiography or computed tomography), patients must be monitored closely for deterioration such as the sudden onset of desaturation, hypotension, sudden tachypnea, and increased oxygen demand. In such cases, interventions such as the placement of chest tubes or pig-tail catheters are required to relieve symptoms and improve hemodynamics and oxygenation [11].

Gattinoni et al. described two phenotypes for COVID-19 pneumonia: the type L and the type H [12]. During the initial course of the disease, there is low elastance, low ventilation-to-perfusion ratio, low lung weight, and low lung recruitability. These are the type L patients who may remain the same clinically for a certain period of time, and then, they either improve or deteriorate. The deterioration leads to high elastance, high right-to-left shunt, high lung weight, and high lung recruitability, which are referred to as type H patients. The type H patients meet all the criteria for severe ARDS. According to Gattinoni et al., type L and H patients are best diagnosed by a CT scan. The other ways to differentiate are respiratory system elastance and recruitability.

In our institution, we had several patients with pulmonary barotrauma of varying severity who were treated based on clinical and radiologic findings. This retrospective study analyzed the incidence of pulmonary barotrauma in patients with COVID-19 pneumonia admitted to our ICU and the overall outcome of these patients.

## Materials and methods

This retrospective study was conducted in Ibra Hospital, North Sharqiya Governorate, Sultanate of Oman. We have observed that some patients admitted with ARDS to the COVID ICU developed P-SILI, and some others developed pulmonary barotrauma as a result of the initiation of respiratory support, which could consist of nasal high-flow oxygenation, non-invasive ventilation (NIV), or invasive mechanical ventilation. After obtaining institutional approval, we retrospectively reviewed data from March 1, 2021, to September 31, 2021. Being a retrospective study, informed consent was not applicable. The data were collected from the Al Shifa patient information portal, which is an electronic medical record system available to all the hospitals in the Ministry of Health, Oman.

We initially used the keyword COVID-19 and obtained a list of all the patients in whom COVID-19 was detected after a positive reverse transcriptase-polymerase chain reaction (RT-PCR). Then, we went through the electronic records of all patients admitted to the ICU and referred to the Department of Anesthesia and Critical Care for respiratory and hemodynamic support. In our hospital, the patients who received supplemental oxygen via masks or nasal cannulae and were hemodynamically stable and not tachypneic or hypoxic were managed by the Department of Internal Medicine. After making a list of all patients who had been referred to our department, we reviewed the records of all patients to determine which of them had developed barotrauma during the admission. Barotrauma was defined as the presence of pneumomediastinum, subcutaneous emphysema, or pneumothorax on imaging. Once we retrieved the list, we have created a database containing the following variables in a Microsoft Excel spreadsheet (Microsoft Corporation, Albuquerque, New Mexico): age, sex, history of smoking, comorbidities, phenotype, type, location, mode of barotrauma, mode of ventilation, duration of ICU stay, interventions performed, and overall outcome (survived/deceased).

Continuous variables were presented as mean with standard deviation or median with an interquartile range as indicated, while the categorical variables were presented as frequency and percentages. The statistical tests were performed using the online statistical tool GraphPad (https://www.graphpad.com/).

## Results

**Figure 1:**
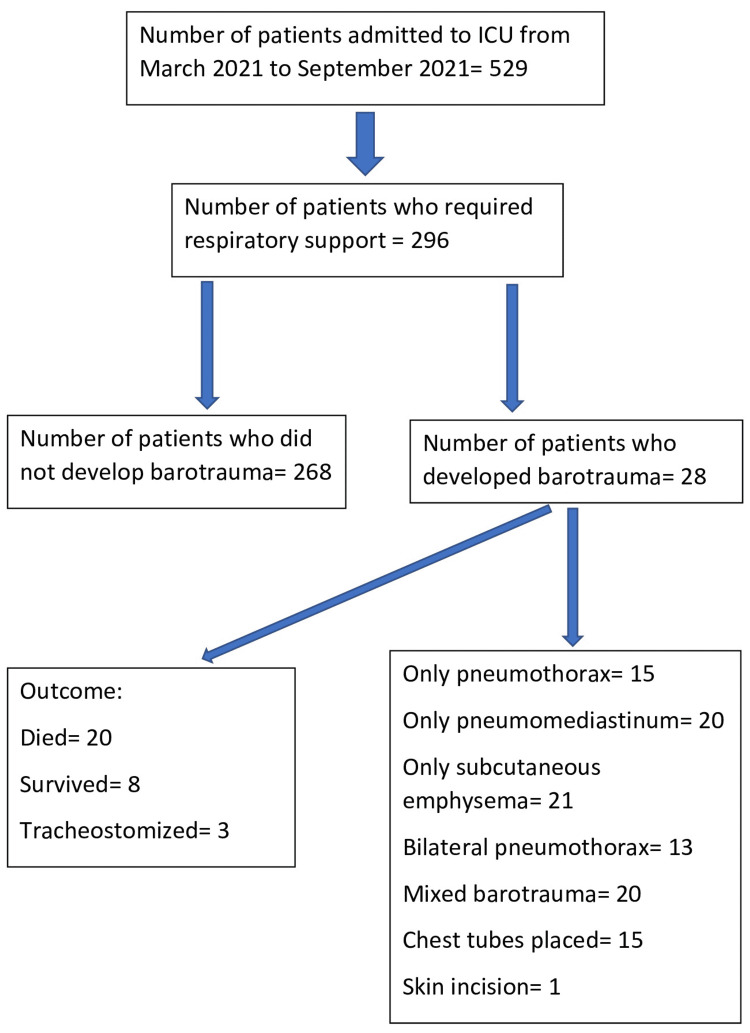
Schematic representation of the patients admitted, who developed barotrauma, the interventions done, and the outcomes ICU: Intensive care unit.

A total of 529 patients were admitted to the COVID ICU during the period from March 2021 to September 2021. The number of patients who required respiratory support for ARDS was 296. Twenty-eight patients developed some form of barotrauma during their stay in the ICU. Figure 1 shows a schematic representation of the patients admitted, who developed barotrauma, the interventions performed, and the outcomes. The demographic details (age, sex, and comorbidities) are shown in Table 1.

**Table 1:** Demographic data, comorbidities, types of barotraumas, ventilatory support used, interventions performed, and the outcomes COPD: Chronic obstructive pulmonary disease; NIV: Non-invasive ventilation; ICU: Intensive care unit; HFNC: High-flow nasal cannula; NRBM: Non-rebreathing mask.

Variables	Patients with barotrauma (n = 28)
Age (years)	47.5 (39.5-66)
Gender (Male/Female)	21/7 (75/25%)
Comorbidities	
Hypertension	3
Diabetes mellitus	4
Ischemic heart disease	2
Cerebrovascular disease	1
Bronchial asthma/COPD	3
Epilepsy	1
Pregnancy	1
None	11
Smoking	None
Types of barotrauma	
Spontaneous	5 (17.85%)
Ventilator-induced	23 (82.14%)
NIV	14 (60.86%)
Invasive ventilation	9 (39.13%)
Pneumothorax	15
Right	7
Left	8
Bilateral	13
Pneumomediastinum	20
Subcutaneous emphysema	21
Neck	21
Chest	21
Abdomen	3
Mixed	20
Respiratory support	
NRBM	5
NIV	7
HFNC	1
Invasive ventilation	9
Interventions done	
Chest tube placement	15
Skin incision	1
Number of days in ICU	19.5 (12.5-26.5)
Number of days for the survivors	27 (18.5-56.5)
Number of days for the patients who died	16.5 (12-24.5)
Outcomes	
Survived	8 (28.57%)
Died	20 (71.42%)
Infection	
Bacterial	22
Fungal	6
Infection and death	17
No infection and death	03
Infection and survival	05
No infection and survival	02
Tracheostomy	3

**Figure 2:**
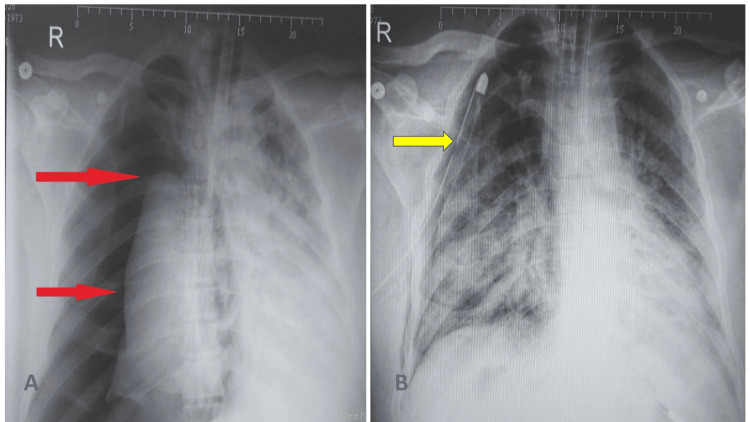
(A) Tension pneumothorax in a mechanically ventilated patient and (B) chest radiograph showing the placement of the chest tube The red arrows in Panel A indicate the pneumothorax, and the yellow arrow in Panel B indicates the chest tube.

**Figure 3:**
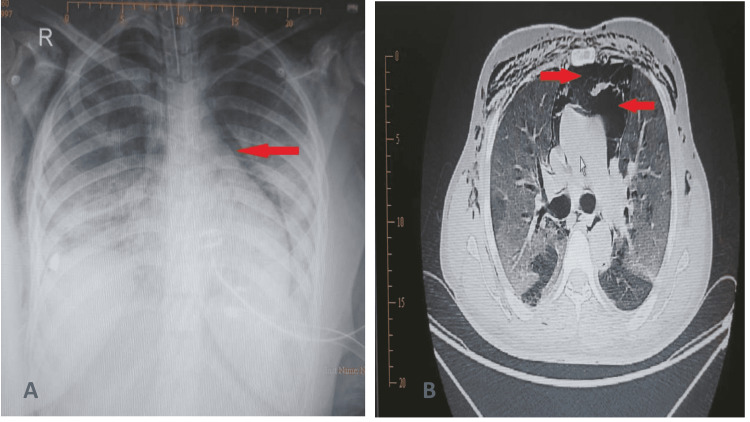
Pneumomediastinum on (A) chest radiograph and (B) axial chest computed tomography The red arrows point to the pneumomediastinum.

A total of 58 barotrauma events occurred in 28 patients (Table 1). P-SILI was observed in five (17.85%) patients, i.e., using a non-rebreathing mask, and 23 (82.14%) patients were ventilator-induced (invasive/non-invasive) (Table 2). Among these 23 patients, 14 patients developed barotrauma after initiating NIV and nine patients after initiating invasive ventilation. Pneumothorax was observed in 15 patients out of which seven were on the right side and eight on the left, and 13 patients presented with bilateral pneumothorax. Fifteen chest tubes were placed for pneumothorax (Figure 2, Panels A and B). Multiple tension-relieving skin incisions were performed on the patient in extensive subcutaneous emphysema. Pneumomediastinum was observed in 20 patients and was mostly located in the chest (21 patients) and neck (21 patients) and over the abdomen in three patients (Figure 3, Panels A and B).

**Table 2:** Ventilatory mode, parameters, and PaO2/FiO2 of 23 patients PSIMV: Pressure-synchronized intermittent mandatory ventilation; NIV: Non-invasive ventilation; PEEP: Positive end-expiratory pressure; FiO_2_: A fraction of inspired oxygen; PaO_2_/FiO_2_: The ratio of partial pressure of oxygen in arterial blood gas with a fraction of inspired oxygen.

Patient number	Mode of ventilation	Fio_2_	Pressure control/pressure support	PEEP (cm of water)	Peak inspiratory pressure (cm of water)	PaO_2_/FiO_2_ ratio
1	PSIMV	55	16	10	15	129
2	NIV	100	10	12	23	51
3	PSIMV	50	20	8	28	130
4	PSIMV	70	20	12	33	80
5	NIV	80	10	14	24	65
6	PSIMV	95	20	10	31	65
7	NIV	90	0	8	11	70
8	NIV	75	3	13	32	80
9	PSIMV	65	30	6	37	96
10	NIV	100	0	10	13	51
11	NIV	80	6	12	20	75
12	PSIMV	80	18	12	30	75
13	NIV	80	0	10	11	65
14	NIV	65	0	2	12	45
15	NIV	90	0	12	15	53
16	PSIMV	60	16	0	26	84
17	NIV	80	0	10	12	75
18	PSIMV	100	12	15	28	66
19	PSIMV	60	26	10	36	110
20	NIV	60	7	10	18	84
21	NIV	70	0	14	17	70
22	NIV	45	12	10	24	90
23	NIV	50	0	15	15	104

The median number of days in the ICU was 19.5 (IQR: 12.5-26.5). Of the 28 patients, eight patients survived and were discharged from the hospital. However, the remaining 20 patients eventually died due to various complications. Out of the five patients who survived, three patients required tracheostomy due to difficult weaning from mechanical ventilation.

## Discussion

In this study, we have shown our findings of pulmonary barotrauma in patients with COVID-19 ARDS. Out of 296 patients, 28 patients developed barotrauma with a total of 58 barotrauma events. Twenty of these patients died eventually (71.42%).

Critically ill patients with decreased lung compliance on positive pressure ventilation are at the risk of over-distension of the relatively preserved parts of the lungs. The more compliant alveoli get inadvertently stretched leading to rupture, which if continued eventually manifests as pulmonary barotrauma [13]. Barotrauma during mechanical ventilation (invasive or non-invasive) presents as pneumomediastinum, subcutaneous emphysema, and pneumothorax. Pneumomediastinum, also known as mediastinal emphysema, is the presence of air tracking along with the mediastinal structures [14,15]. The pathophysiology of pulmonary barotrauma is based on the Macklin phenomenon, which describes the occurrence of a large pressure gradient between the marginal alveoli and the lung interstitium, resulting in air leakage to the surrounding bronchovascular sheath [16-19]. Lemmers et al. hypothesized that the pneumomediastinum and subcutaneous emphysema in COVID-19 patients could be due to increased lung frailty that develops in COVID ARDS. This was based on their experience with 23 pulmonary barotrauma patients admitted to their ICU [20].

On several occasions, the pressure in the mediastinum is relieved by the air tracking to the subcutaneous tissue, resulting in subcutaneous emphysema (SCE), which is detected in 70% of patients with pneumomediastinum. SCE presents as palpable crepitus, and the most common site is the root of the neck [21]. This is because the visceral layer of the deep cervical fascia is in continuity with the mediastinum. Once released, the air travels to the face, limbs, abdomen, and perineum owing to the connection among the facial planes. Although the release of air in the subcutaneous tissue relieves the mediastinal pressure, it may lead to pneumopericardium, pneumorrhachis (air in the spinal canal), and pneumothorax [22,23].

Barotrauma was a relatively common finding in patients with COVID-19 pneumonia during the pandemic, which was either P-SILI or ventilator-induced (invasive or non-invasive) [24,25]. Many researchers suggested that spontaneous pneumothorax was possible in patients with high C-reactive protein, severe lymphopenia, high D-dimer, high lactate dehydrogenase and ferritin, or high viral load [26,27].

Pneumothorax is air entrapment in the pleural space that can occur spontaneously or following trauma. Spontaneous pneumothorax can be primary or secondary. Primary pneumothorax occurs in the absence of underlying parenchymal lung diseases, whereas secondary pneumothorax affects diseased lungs as in ARDS, including COVID-19 ARDS. Pneumothorax is considered a medical emergency, especially if it is a tension pneumothorax, and therefore prompt identification and management are essential to prevent life-threatening events [28,29].

Gorospe et al. reported four patients with COVID-19 pneumonia who developed spontaneous pneumomediastinum (58-65 years of age, two men and two women) [30]. As per the article, all patients had severe cough due to pneumonia, which led to an alveolar rupture and secondary gas leakage to the peribronchovascular pulmonary interstitium, thereby causing pneumomediastinum. Barotrauma was reported before starting any kind of invasive or NIV in all four patients.

Edwards et al. reported pulmonary barotrauma in a case series involving 13 patients who were mechanically ventilated [31]. Three patients out of 13 had pneumothoraces and pneumomediastinum, 12 patients had pneumomediastinum and SCE, and one patient had pneumothorax alone. The average days on the ventilator were 3.4, the average positive expiratory-end pressure was 15.5 cm H_2_O, the dynamic compliance was 33.8 mL/cm H_2_O, and the P/F ratio was 165. Four patients underwent chest tube insertion, and two patients had pig-tail catheter placement done.

Shaikh et al. reported five patients with COVID-19 ARDS on mechanical ventilation on moderate positive end-expiratory pressure (PEEP) who developed tension pneumomediastinum [32]. In this series, two patients required chest drain to relieve the tension, one patient was managed with extracorporeal membrane oxygenation (ECMO) because of severe ARDS, and two patients died eventually. The authors concluded that the management in such situations should involve prompt recognition and decompression with the insertion of drains in unstable situations, whereas stable patients can be closely monitored.

Coppola et al. reported their experience in six cases of pneumomediastinum and one case of pneumothorax during treatment in a sub-intensive area of the division of medicine [33]. In this series, four patients had pneumomediastinum and SCE following NIV. All were managed conservatively. One patient died following a massive myocardial infarction, and one patient had a chest tube insertion due to extensive pneumothorax. Ekanem et al. reported that in their institute, out of 1,619 COVID-19 patients admitted, 22 patients (1.4%) developed spontaneous pneumothorax during their hospitalization without evidence of traumatic injury [34]. Sixteen of the 22 patients underwent chest tube placement based on the magnitude of barotrauma and associated symptomatology. Half of the number of patients were not on mechanical ventilation when pneumothorax was diagnosed in four patients (18%) only on nasal cannula.

Talan et al. reported nine mechanically ventilated patients out of 161 admitted patients who had a pneumothorax and/or pneumomediastinum during their stay in the ICU with COVID-19 pneumonia [35]. All patients were managed conservatively by limiting their PEEP and maximum inspiratory pressures and were followed by daily chest radiographs for detection of any progress. Out of the nine patients, one patient survived and was discharged, whereas the other succumbed to various complications. Hemodynamic instability due to pneumothorax and/or pneumomediastinum was not observed in any of the patients. Neither was tension pneumothorax seen in any of the patients. The most common reason for death was caused by serious bacterial infections resulting in septic shock. Volpi et al. published their series of three cases (52 year/male, 68 year/male, and 66 year/male) who were mechanically ventilated because of severe ARDS [36]. All patients developed extensive pneumothorax and pneumomediastinum, with SCE but were managed conservatively by meticulous monitoring and daily chest radiographs and did not eventually need chest drains. The authors emphasized close monitoring and suggested using invasive procedures based on the clinical picture and hemodynamic stability.

In a retrospective case-control study by Venkateswaran et al., the authors compared the characteristics, associations, and outcomes of COVID-19 patients admitted to the ICU with and without barotrauma [37]. The number of patients admitted over three months was 827, out of which 30 patients (3.6%) developed barotrauma of various types (pneumothorax was most common, in 26 out of 30 patients). On analysis, the authors concluded that the duration of mechanical ventilation, the length of ICU stay, and the mortality rate were significantly higher in patients who developed barotrauma when compared to the non-barotrauma group of patients. In a systematic review and meta-analysis by Shrestha et al., the authors identified and analyzed 15 observational studies and 118 case reports/series. On analysis, the authors concluded that the incidence of barotrauma was 4.2% (2.4%-7.3%) among hospitalized patients; 15.6% (11-21.8%) among critically ill patients; and 18.4% (13-25.3%) in patients on invasive mechanical ventilation. This demonstrated a linear relationship of barotrauma with the severity of the underlying disease [38].

Any sudden respiratory and/or hemodynamic deterioration in a patient with COVID-19 ARDS should raise the suspicion of barotrauma, i.e., pneumothorax or pneumomediastinum with a differential diagnosis of pulmonary embolism or exacerbation of ARDS. The attending clinician should request imaging studies, electrocardiogram (ECG), arterial blood gas analysis, and D-dimer to arrive at the diagnosis [39]. Although anecdotal, few studies suggested that the episodes of coughing that increase the pressure in the chest, positive pressure ventilation, prone position therapy, and steroid therapy may contribute to the development of pneumomediastinum and pneumothorax. Most of the patients have a spontaneous resolution with conservative management, which includes close monitoring, bed rest, analgesia, adequate nutrition, and oxygen therapy. Invasive methods should be reserved for hypoxic patients with or without hemodynamic compromise with deterioration not resolving with conservative measures [40,41]. In our series, the mortality of patients having barotrauma was 71.42% in the ICU. This was similar to the previously published case series and observational studies.

There are several limitations of this study. First, due to its retrospective nature, this study has issues such as missing data and selection bias. We have collected the data from the electronic patient management portal, but still, there is a possibility of missing entries. Second, the number of patients included in this study is limited, and therefore the conclusions are not generalizable. Third, it is a single-center study with the involvement of different clinicians attending the patients on a rotation basis. There were no standard protocols followed in offering respiratory support. It was based on the discretion of the attending anesthesiologist and intensivist.

## Conclusions

Even while using lung-protective ventilation, patients with COVID-19 ARDS could develop pulmonary barotrauma. These patients require meticulous monitoring and adequate sedation. If sudden deterioration is observed in the form of desaturation, hypotension, or unexplained high airway pressures, radiological imaging should be performed, and based on this, a decision can be made whether close monitoring or chest drainage (for pneumothorax) is required. Daily clinical examination should also involve examining the chest, axilla, and neck for any evidence of SCE, which is suggestive of ongoing pulmonary barotrauma.
